# *QuickStats:* Percentage* of Children and Adolescents Aged 5–17 Years Who Had Chronic School Absenteeism Due to Illness, Injury, or Disability During the Past 12 Months,^†^ by Age Group and Year — National Health Interview Survey,^§^ United States, 2019 and 2022

**DOI:** 10.15585/mmwr.mm7308a6

**Published:** 2024-02-29

**Figure:**
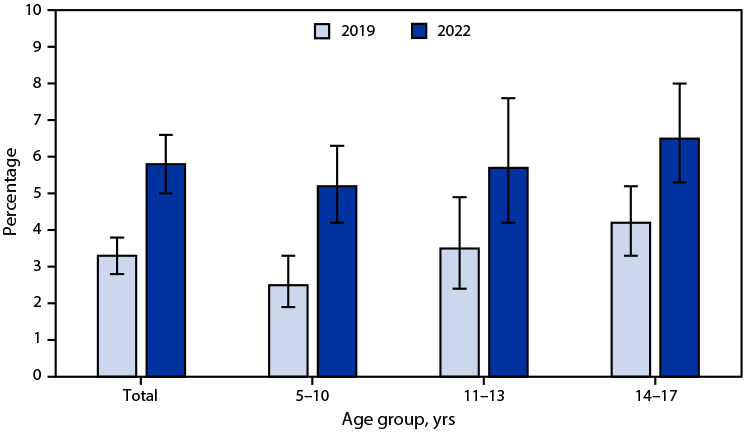
The percentage of children and adolescents aged 5–17 years who had chronic school absenteeism during the past 12 months was higher in 2022 (5.8%) than in 2019 (3.3%). From 2019 to 2022, the percentage of children who had chronic school absenteeism increased for each age group. The percentage of children who had chronic school absenteeism increased with increasing age in 2019; no significant differences by age occurred in 2022.

**Source:** National Health Interview Survey, 2019 and 2022. https://www.cdc.gov/nchs/nhis.htm

